# The Association between Job-Related Psychosocial Factors and Prolonged Fatigue among Industrial Employees in Taiwan

**DOI:** 10.1371/journal.pone.0150429

**Published:** 2016-03-01

**Authors:** Feng-Cheng Tang, Ren-Hau Li, Shu-Ling Huang

**Affiliations:** 1 Department of Occupational Medicine, Changhua Christian Hospital, Changhua, Taiwan; 2 Department of Leisure Services Management, Chaoyang University of Technology, Taichung, Taiwan; 3 Department of Psychology, Chung-Shan Medical University, Taichung, Taiwan; 4 Room of Clinical Psychology, Chung-Shan Medical University Hospital, Taichung, Taiwan; Kaohsiung Chang Gung Memorial Hospital, TAIWAN

## Abstract

**Background and Objectives:**

Prolonged fatigue is common among employees, but the relationship between prolonged fatigue and job-related psychosocial factors is seldom studied. This study aimed (1) to assess the individual relations of physical condition, psychological condition, and job-related psychosocial factors to prolonged fatigue among employees, and (2) to clarify the associations between job-related psychosocial factors and prolonged fatigue using hierarchical regression when demographic characteristics, physical condition, and psychological condition were controlled.

**Methods:**

A cross-sectional study was employed. A questionnaire was used to obtain information pertaining to demographic characteristics, physical condition (perceived physical health and exercise routine), psychological condition (perceived mental health and psychological distress), job-related psychosocial factors (job demand, job control, and workplace social support), and prolonged fatigue.

**Results:**

A total of 3,109 employees were recruited. Using multiple regression with controlled demographic characteristics, psychological condition explained 52.0% of the variance in prolonged fatigue. Physical condition and job-related psychosocial factors had an adjusted R^2^ of 0.370 and 0.251, respectively. Hierarchical multiple regression revealed that, among job-related psychosocial factors, job demand and job control showed significant associations with fatigue.

**Conclusion:**

Our findings highlight the role of job demand and job control, in addition to the role of perceived physical health, perceived mental health, and psychological distress, in workers’ prolonged fatigue. However, more research is required to verify the causation among all the variables.

## Introduction

Fatigue is a common complaint of the working population. A recent study found that at least 19% of healthy workers reported experiencing fatigue, heavy-headedness, headache, dizziness, or difficulty in concentrating [1]. Among employees visiting a general practitioner, one in seven employees (13.9%) scheduled the appointment due to fatigue-related problems [2]. Acute fatigue at work, defined as a sudden onset of physical and mental exhaustion after a short-term stressful work situation, is a normal experience that disappears after a period of rest or when tasks are changed. In contrast, workers do not easily recover from chronic fatigue in the short term. Although no perfect definition of chronic fatigue exists, it is usually regarded as long-continued fatigue not relieved by rest and lasting for a period ranging from weeks to months. In the present study, prolonged fatigue is defined as the extent of subjective fatigue experienced during the past two weeks. Several studies have also adopted this definition [3,4]. A large-scale survey using a heterogeneous working sample showed a 22% prevalence of prolonged fatigue in workers [4]. Chronic fatigue may lead to a global condition of physical dysfunction among workers and put the individual at risk for subsequent sick leave, work disability or intentions to leave [3,5,6]. Moreover, individuals with chronic fatigue who receive no treatment have a poor prognosis [7]. Clearly, the issue of fatigue should therefore attract more attention in occupational health research. It should be noted that the definitions and the levels of fatigue varied in the above-mentioned studies.

A continuous distribution of fatigue scores was found in the working population [4,8] and in a clinical population [9]. Fatigue is best viewed as a continuum rather than as a dichotomy [10]. The etiology of fatigue is likely multidimensional and exists inside and outside the workplace. Fatigue includes physical, emotional, behavioral, and cognitive components [7,10,11]. Individuals’ physical and psychological conditions were found to be associated with fatigue [10,12]. Physical inactivity was regarded as one of the perpetuating factors of fatigue [7]. Psychological distress was also associated with fatigue [4]. Therefore, perceived physical health, exercise routine, perceived mental health, and psychological distress should be considered when trying to better understand the related determinants of fatigue among workers.

In previous studies, task factors and sleep-related factors were frequently discussed work-based contributors to fatigue [13,14]. Monotonous or repetitive work, long working hours, and the type (day or night), pattern (permanent or rotating), and duration of the work shift were found to be associated with fatigue. Inadequate elements of the work environment, such as light, temperature, humidity, sound, and ventilation, were also risk factors for work-related fatigue [1,15]. In addition, fatigue was found to be connected with “presenteeism,” the phenomenon of employees staying at work when they should be on sick leave and usually causing a reduction in productivity [16,17]. The American Commission of Occupational and Environmental Medicine has published management guidelines concerning fatigue in the workplace with the goal of expanding the skills and resources to design and implement a fatigue-risk-management system [15]. However, existing knowledge pertaining to the relationship between job-related psychosocial factors and prolonged fatigue was limited. Uncertainty and instability exist widely in today’s work environment, leading to increased stress for employees [18]. Job-related psychosocial factors are therefore concerns that need to be addressed. The Job Demand–Control model has been widely used to assess psychosocial factors in the workplace [19]. Job demand, job control (decision latitude and skill discretion), and social support at work were proposed as psychosocial characteristics of a job that could be used to predict the outcomes of health [20]. In the limited literature, low job control and low social support at work were found to be associated with prolonged fatigue in employees across the trades [21]. In a study of Chinese nurses, chronic fatigue was predicted solely by job demand rather than job control or workplace social support [22]. The mixed findings may be due to the different definitions and measures of fatigue.

Most studies found that fatigue was more prevalent in women than in men [7,8,23,24], though a few studies reported no gender difference [4,9]. In previous studies, the association between age and fatigue was not consistent [8,24], with generally only weak associations between them being found [7,9]. Furthermore, some research reported that the associations differed by gender [4,23]. Gender and age were therefore suggested for consideration as covariates calling for statistical control in analyses relating to the research on fatigue [4]. In addition, because occupation determines work content, socioeconomic status, ways of living, and so on, occupation may need to be considered when exploring employees’ level of prolonged fatigue.

In the present study, the significances of physical condition (perceived physical health and exercise routine), psychological condition (perceived mental health and psychological distress), and job-related psychosocial factors (job demand, job control, and workplace social support) in relation to prolonged fatigue would be examined individually and hierarchically among industrial employees in Taiwan. The aims of the study were (1) to assess the individual relations of physical condition, psychological condition, and job-related psychosocial factors to prolonged fatigue, respectively, after controlling demographic characteristics, and (2) to clarify the associations between job-related psychosocial factors and prolonged fatigue using multiple regression in hierarchical ways, when demographic characteristics, physical condition, and psychological condition were considered simultaneously.

## Methods

### Recruitment and Ethical Approval

The study adopted a cross-sectional research method with convenient sampling. Some principles for sampling were as follows: First, the industrial factory, the main worksite for Taiwanese employees [25], was the target. Second, factories willing to cooperate with the researchers were eligible for this study. This allowed the study to proceed without difficulties. Furthermore, the occupational safety personnel in these factories were willing to distribute and collect the survey questionnaires. In addition, to represent a variety of employees, different-sized factories, diverse activity types, and the workers’ demographic characteristics were considered. Two small/medium-scale factories (fewer than 300 employees) and four large-scale factories (300 or more employees) in central Taiwan were eventually recruited. Their principal activities included transportation and manufacturing of electronic components, food, motor vehicle parts, paper containers, and transport equipment. A total of 5,204 full-time Taiwanese employees, sharing the same language and culture, were invited to participate in the study. All potential subjects were requested to complete a self-administrated questionnaire anonymously and to contribute their opinions on worksite-health-promotion planning. Among these full-time employees, 3,589 workers (69.0% of all eligible participants) agreed to take part voluntarily in the study. Participants received a consent form that they were required to read. Written consent was not required because the data were analyzed anonymously. The study was conducted according to the Declaration of Helsinki and was approved by the Institutional Review Board of the Changhua Christian Hospital in Taiwan (IRB serial number: 110508).

### Measures

The survey questionnaire comprised five parts: demographic characteristics, physical condition, psychological condition, job-related psychosocial factors, and prolonged fatigue. Demographic data included gender, age, and occupation. Occupation was divided into three categories: management, white-collar worker (including professional, technician, office worker, and service worker), and blue-collar worker (including crafts worker and machine operator) [26].

#### Physical condition

Physical condition included perceived physical health and whether the individuals exercised regularly. Using a 5-point Likert response format ranging from “very poor” to “excellent,” perceived physical health was measured by one question: “How would you rate your overall physical health at the present time?” The exercise habit was measured by the following question: “Do you do a moderate level of exercise for at least 30 minutes every day?” The answer was a dichotomous “yes” or “no.”

#### Psychological condition

Psychological condition included the variables of perceived mental health and psychological distress. The following single question was used with a 5-point Likert response format to obtain input on perceived mental health: “How would you rate your overall mental health at the present time?” Psychological distress was measured by the Chinese Health Questionnaire (CHQ-12), a well-validated instrument [27]. The CHQ-12 was translated from the 12-item General Health Questionnaire [28] with culturally relevant modifications. The twelve items assessed the extent of depression, anxiety, sleep disturbance, somatic concerns, and interpersonal difficulties. The CHQ-12 was on a 4-point Likert scale ranging from zero to three. A higher total score presented a higher level of psychological distress. For this study, alpha reliability was 0.89.

#### Job-related psychosocial factors

The Chinese version of the JCQ-like questionnaire based on a 4-point Likert scale was adopted in this study to determine the levels of job demand (five items), job control (nine items), and social support at work (four items pertaining to supervisor support and four items pertaining to coworker support) [29]. This scale was shortened from the comprehensive Job Content Questionnaire (JCQ), which, developed by Karasek [30], has become one of the most popular instruments for assessing psychosocial factors in the workplace. For each subscale, a sum of weighted-item scores was calculated according to existing recommendations [29,31]. The psychometric characteristics of the Chinese version of the JCQ-like questionnaire were reported with 0.55–0.86 of Cronbach’s alpha coefficients and four empirical factors (including job demand, job control, supervisor support, and coworker support), which corresponded with theoretical constructs when used for Taiwanese workers [29]. A higher summed score represented a higher level of job demand, job control, or workplace social support. In the present study, Cronbach’s alpha coefficients were 0.66, 0.71, and 0.86 for the subscales of job demand, job control, and workplace social support, respectively.

#### Prolonged fatigue

The Checklist Individual Strength questionnaire (CIS) [3] was developed to assess the extent of the subjective fatigue, reduction in motivation, reduction in activity, and reduction in concentration during the two-week period. The validity and reliability of the Chinese version of the CIS were adequate when applied to the Taiwanese population [32]. For delimitating the research purpose, only the subscale of subjective fatigue (eight items) was adopted in the present study to measure the level of prolonged fatigue, and its Cronbach’s alpha coefficient was 0.90 in the present study. The questionnaire was on a 7-point Likert scale ranging from one to seven. A higher summed subscale score indicated a higher level of prolonged fatigue.

### Statistical Analysis

The SPSS-13.0 software for Microsoft Windows was used for statistical analysis. The demographic characteristics of the participants, along with their data pertaining to physical, psychological, and job-related psychosocial factors, were summarized using descriptive statistics. The Chi-square test or F-test was adopted to compare the differences in demographic characteristics and other surveyed variables by occupation. Pearson’s correlation was used to analyze the relationships between the continuous variables. Multiple linear regression was adopted, with age, gender, and occupation controlled, to investigate the individual relations of physical condition, psychological condition, and job-related psychosocial factors to prolonged fatigue. Among the controlled factors, gender and occupation have been transformed into dummy variables beforehand. In order to further clarify the comparative effects of physical condition, psychological condition, and job-related psychosocial factors on prolonged fatigue, multiple linear regression in hierarchical ways was adopted. In model 1, the demographic characteristics of gender, age, and occupation were first put in the regression as independent variables. In model 2, physical and psychological variables were added to explore their relation to prolonged fatigue. Finally, in model 3, job demand, job control, and workplace social support were added simultaneously in order to determine the unique significances of these job-related psychosocial factors.

## Results

Of the 3,589 questionnaires distributed, 3,109 were successfully collected, representing a response rate of 86.6%. Table 1 lists the demographic characteristics, physical condition, psychological condition, job-related psychosocial factors, and prolonged fatigue of participants by occupation. Of the participants, 81.9% were males and 18.1% were females. The average age was 44.8 years (SD = 8.9). The majority of the participants were white-collar workers (63.9%), followed by blue-collar workers (27.9%). Management had the fewest in number (n = 253, 8.2%), but the highest average age, at 48.0 years (SD = 6.2). The comparisons of gender, age, perceived physical health, perceived mental health, psychological distress, all the job-related psychosocial factors, and prolonged fatigue by occupation showed significant differences. Management showed higher scores on perceived physical health, perceived mental health, job demand, job control, and workplace social support, as well as lower scores on psychological distress and prolonged fatigue compared to white-collar and blue-collar employees. The only variable that did not reach a statistical significance was regular physical exercise. This indicated that most of the employees, regardless of occupation, did not exercise regularly.

**Table 1:** Demographic characteristics, physical, psychological, and job-related psychosocial variables of all participants.

Variables	Total (n = 3109) n(%)^a^	Management (n = 253) n(%)^a^	White-collar (n = 1964) n(%)^a^	Blue-collar (n = 857) n(%)^a^	χ^2^
Gender					99.22***
Male	2534 (81.9)	227 (90.1)	1503 (76.8)	778 (91.5)	
Female	559 (18.1)	25 (9.9)	454 (23.2)	72 (8.5)	
Perceived physical health					18.77*
Excellent	156 (5.1)	19 (7.5)	104 (5.3)	30 (3.5)	
Good	1141 (36.9)	106 (42.1)	732 (37.5)	294 (34.6)	
Ordinary	1537 (49.8)	112 (44.4)	965 (49.4)	442 (52.1)	
Poor	238 (7.7)	15 (6.0)	144 (7.4)	76 (9.0)	
Very poor	16 (0.5)	0 (0.0)	9 (0.5)	7 (0.8)	
Regular exercise					1.90
Yes	656 (21.4)	47 (18.9)	429 (22.1)	174 (20.5)	
No	2415 (78.6)	202 (81.1)	1512 (77.9)	674 (79.5)	
Perceived mental health					36.45***
Excellent	211 (6.8)	29 (11.6)	128 (6.6)	50 (5.9)	
Good	1306 (42.3)	125 (49.8)	846 (43.4)	325 (38.2)	
Ordinary	1389 (45.0)	88 (35.1)	878 (45.0)	406 (47.8)	
Poor	159 (5.2)	9 (3.6)	88 (4.5)	60 (7.1)	
Very poor	20 (0.6)	0 (0.0)	11 (0.6)	9 (1.1)	
Variables (possible range)	Mean (SD)	Mean (SD)	Mean (SD)	Mean (SD)	F
Age in years (18–65)	44.8 (8.9)	48.0 (6.21)	44.3 (9.2)	45.1 (8.6)	20.46***
Psychological distress (0–36)	9.3 (5.3)	8.0 (5.0)	9.1 (5.1)	10.2 (5.6)	20.95***
Job demand (12–48)	30.7 (4.3)	31.1 (4.3)	30.5 (4.3)	30.9 (4.2)	3.64*
Job control (24–96)	64.4 (8.0)	70.6 (6.5)	64.7 (7.9)	61.8 (7.7)	125.77***
Workplace social support (8–32)	23.2 (2.7)	24.0 (1.7)	23.3 (2.7)	22.6 (2.8)	31.41***
Prolonged fatigue (8–56)	26.2 (8.5)	24.2 (8.4)	26.0 (8.4)	27.2 (8.6)	13.80***

^a^ Calculated according to a percentage of the valid count.

*p < .05;

**p < .01;

***p < .001 calculated using χ^2^ or *F*-test.

Table 2 shows bivariate correlations between variables. The correlation coefficients were statistically significant between the variables of physical condition, psychological condition, job-related psychosocial factors, and prolonged fatigue (coefficients ranged from .06 to .68, *p* < .01), with the exceptions of the relationships between regular exercise and some other variables. Among these correlations, prolonged fatigue correlated highly with perceived physical health (r = -.55, *p* < .01), perceived mental health (r = -.58, *p* < .01), and psychological distress (r = .65, *p* < .01). The variable of regular exercise barely correlated with only three variables, namely perceived physical health (r = .04, *p* < .05), perceived mental health (r = .04, *p* < .05), and workplace social support (r = -.04, *p* < .05).

**Table 2:** Correlation matrix of physical, psychological, and job-related psychosocial variables.

Variables	1	2	3	4	5	6	7
1 Perceived physical health	1.00						
2 Regular exercise	.04*	1.00					
3 Perceived mental health	.68**	.04*	1.00				
4 Psychological distress	-.49**	.01	-.58**	1.00			
5 Job demand	-.16**	.01	-.22**	.26**	1.00		
6 Job control	.23**	.01	.28**	-.25**	-.06**	1.00	
7 Workplace social support	.18**	-.04*	.25**	-.24**	-.23**	.41**	1.00
8 Prolonged fatigue	-.55**	.00	-.58**	.65**	.32**	-.32**	-.22**

*p < .05;

**p < .01.

Table 3 shows the summary of the individual contributions of physical condition, psychological condition, and job-related psychosocial factors to prolonged fatigue. Using multiple linear regression with controlled gender, age, and occupation, all variables of physical condition, psychological condition, and job-related psychosocial factors showed significant associations with prolonged fatigue. Psychological condition, when compared to physical condition and job-related psychosocial factors, was the strongest contributor to prolonged fatigue (adjusted R^2^ = 0.520, *p* < .001). Physical condition and job-related psychosocial factors had an adjusted R^2^ of 0.370 and 0.251, respectively (*p* < .001).

**Table 3:** Summary of multiple linear regressions for physical condition, psychological condition and job-related psychosocial factors predicting prolonged fatigue separately, with gender, age, and occupation controlled.

Variables	Β	SE	β	*p*	Adjusted R^2^	F
Physical condition					0.370	296.70***
Perceived physical health	-6.23	0.17	-0.53	<.001		
Regular exercise	0.69	0.30	0.03	.022		
Psychological condition					0.520	548.02***
Perceived mental health	-3.34	0.18	-0.29	<.001		
Psychological distress	0.74	0.03	0.46	<.001		
Job-related psychosocial factor					0.251	142.29***
Job demand	0.54	0.03	0.27	<.001		
Job control	-0.25	0.02	-0.23	<.001		
Workplace social support	-0.21	0.06	-0.07	<.001		

Β denotes unstandardized regression coefficient; SE denotes standard error; β denotes standardized regression coefficient.

***p < .001.

Table 4 shows the results of prolonged fatigue by multiple linear regression in hierarchical ways. In model 1, age and occupation showed significant associations with prolonged fatigue. Younger employees (Β = -0.28, *p* < .001) reported a higher level of fatigue. Blue-collar workers (Β = 2.18, *p* < .001) were at greater risk of prolonged fatigue as compared with management. In model 2, when the variables of physical and psychological conditions were added into the regression, the effect of occupation became nonsignificant. In addition to gender and age, perceived physical health (Β = -2.58), perceived mental health (Β = -1.87), and psychological distress (Β = 0.68) showed significant associations with prolonged fatigue (*p* < .001 for all). In model 3, the adjusted R^2^ increased only 0.025 when job-related psychosocial factors were added. Job demand (Β = 0.28, *p* < .001) and job control (Β = -0.11, *p* < .001) showed significant associations with prolonged fatigue, while workplace social support did not have a significant relationship with prolonged fatigue. In addition, gender, age, perceived physical health, perceived mental health, and psychological distress retained significant associations with prolonged fatigue in this model.

**Table 4:** Summary of hierarchical regression analysis for variables predicting prolonged fatigue.

	Model 1				Model 2				Model 3			
Variables	Β	SE	β	*p*	Β	SE	β	*p*	Β	SE	β	*p*
(constant)	38.39	1.01		<.001	18.41	0.97		<.001	17.57	1.82		<.001
Gender (man)^a^	0.50	0.39	0.02	.200	0.77	0.28	0.04	.006	0.68	0.28	0.03	.016
Age	-0.28	0.02	-0.30	<.001	-0.18	0.01	-0.19	<.001	-0.17	0.01	-0.18	<.001
Occupation (white-collar)^b^	0.72	0.55	0.04	.187	-0.22	0.40	-0.01	.575	-0.51	0.40	-0.03	.196
Occupation (blue-collar)^b^	2.18	0.58	0.12	<.001	-0.07	0.42	-0.00	.863	-0.62	0.43	-0.03	.145
Perceived physical health					-2.58	0.20	-0.22	<.001	-2.49	0.20	-0.21	<.001
Regular exercise					0.39	0.26	0.02	.124	0.39	0.25	0.02	.119
Perceived mental health					-1.87	0.21	-0.16	<.001	-1.60	0.21	-0.14	<.001
Psychological distress					0.68	0.03	0.42	<.001	0.61	0.03	0.38	<.001
Job demand									0.28	0.03	0.14	<.001
Job control									-0.11	0.02	-0.11	<.001
Workplace social support									0.03	0.04	0.01	.533
Full model, F	82.07	***			449.95	***			351.70	***		
Full model, Adjusted R^2^	0.096				0.545				0.570			

^a^ woman as a reference;

^b^ management as a reference;

***p <.001.

## Discussion

This study aimed to assess the relation of physical condition, psychological condition, and job-related psychosocial factors to prolonged fatigue, respectively, among industrial employees in Taiwan. This study also explored the associations between job-related psychosocial factors and prolonged fatigue when demographic data, physical condition, and psychological condition were controlled. The results showed that 52.0% of the variance of prolonged fatigue among employees could be explained by psychological condition (perceived mental health and psychological distress), which represented the largest influence compared with physical condition and job-related psychosocial factors. In addition, job demand, job control, and workplace social support had individually significant associations with prolonged fatigue. However, in the hierarchical regression, among the job-related psychosocial factors, only job demand and job control showed significant associations with prolonged fatigue when all other variables were taken into account.

Perceived physical health was found to be negatively associated with prolonged fatigue in both individual and hierarchical analyses. This result is consistent with previous studies’ findings. In a population-based adult survey, poor self-perceived health was significantly associated with reported fatigue [33]. Poorer health perception was also found to be associated with higher levels of chronic fatigue in young people employed during the school year [34]. In terms of exercise, graded exercise therapy is regarded as an effective rehabilitation strategy for chronic fatigue [7]. Evidence of an altered immune response to exercise in patients with chronic fatigue syndrome was supported [35]. However, in the healthy population, whether regular exercise could contribute to lower levels of prolonged fatigue was seldom studied. Only a few studies mentioned that lack of exercise was associated with fatigue [36,37]; nevertheless, the effect of regular exercise on prolonged fatigue was not found in the present study by advanced statistical analysis. A possible explanation for the conflict was that the type (aerobic versus nonaerobic) and the intensity of exercise were not classified in detail in these studies. Notably, increasing perceived exercise exertion was also associated with increasing symptoms of chronic fatigue [38]. An exact definition of physical activity is therefore recommended for future studies in this field.

Psychological distress and perceived mental health were strongly associated with the level of prolonged fatigue in both individual and hierarchical analyses. In particular, psychological distress showed greater influence than did perceived mental health. These findings are consistent with other research. Psychological distress can be seen as a precipitating factor of unexplained fatigue [7]. A previous study found that fatigue was associated with higher depression scores in workers in southern Taiwan [36]. In a survey of employees consulting a general practitioner, the participants whose visit was fatigue related also had higher levels of fatigue and more mental health problems than those whose visit was non-fatigue related [2]. In a population-based survey, Chinese individuals with chronic fatigue reported poorer mental health [39]. Previous studies also found that the most commonly cited reasons for fatigue were psychosocial problems; fatigue and psychological morbidity correlated closely [7,8,21]. Psychological factors should not be neglected when developing health-promoting programs to prevent workers from experiencing fatigue. Strategies to enhance workers’ mental health may also be useful for alleviating the level of fatigue; more research is required to confirm the effects of the strategies.

A large-scale Belgian survey found that job demand instead of job control showed a significant effect on persistent fatigue; however, the measurement of fatigue was based on a single item [40]. Another large-scale study focused on a heterogeneous working population revealed different results [21]. In that study, low job control and low social support at work were found to be significantly associated with fatigue, after an adjustment for psychological distress. Similarly, a study carried out in Taiwan showed that workers in the fatigued group were more likely to have lower job control and less social support at work than their non-fatigued counterparts, even though the levels of job demand in the two groups were not significantly different [36]. In the present study, both higher job demand and lower job control were found to be related to prolonged fatigue. Workplace social support did not show a significant relation to prolonged fatigue in regression with hierarchical type (Table 4); however, it showed a significant association with prolonged fatigue when job-related psychosocial variables with controlled demographic characteristics were solely taken into account (Table 3). The inconsistent results among these mentioned studies might be mainly due to different definitions and measures of fatigue and whether the manifest covariates, such as physical and psychological conditions, were considered in the research. Moreover, these studies did not take into account the work environment and the occurrence of medical conditions correlated with workers’ fatigue. Studies exploring the relationship between prolonged fatigue and job-related psychosocial factors were insufficient. More research and new hypotheses are recommended to corroborate the causation. At the current stage, among the job-related psychosocial factors, job demand and job control should not be overlooked when planning workplace health-promoting programs to mitigate workers’ fatigue.

In the present study, when compared with white-collar and blue-collar workers, management reported greater perceived physical health, greater perceived mental health, lower levels of psychological distress, higher job control, higher levels of social support at work, and lower levels of prolonged fatigue. This occupation-related information seemed highly meaningful. However, the effect of occupation on prolonged fatigue became nonsignificant after adjustment for other variables. Previous studies supported this finding of nonsignificance. Evengard et al. reported that chronic fatigue was more prevalent in individuals in certain occupational categories but that this might be due to confounding by gender [41]. Helfenstein et al. also indicated that scientific evidence did not support the proposition that some specific fatigue-related problems might be caused by occupational issues [42]. The occupational category therefore may not need to be emphasized in workplace health promotion for mitigating prolonged fatigue if workers’ physical health, mental health, and job-related psychosocial factors have been considered.

Only a limited number of studies focus on the association between prolonged fatigue and job-related psychosocial factors, and even fewer studies view fatigue as a continuum and address its multidimensional etiologies. The present study adopted a multidimensional perspective, including physical, psychological, and job-related psychosocial factors, as well as a large sample size to explore prolonged fatigue among workers. However, the present study had several limitations. First, this study did not consider the participants’ work environment and the occurrence of medical conditions correlated with fatigue. Therefore, some potential biases may exist. Second, a certain number of eligible participants chose not to participate in the present study, and we were not sure whether this group was missing at random (MAR). Moreover, because data were obtained anonymously, nonresponse bias could not be investigated. Third, all the scales used in the present study were self-reported. Response bias, overestimation, or underestimation may have produced confounded results. Fourth, the low reliability associated with job demand and job control may have restricted the relationships between job-related psychosocial factors and prolonged fatigue. Future research on prolonged fatigue and the effort-reward imbalance (ERI) model developed by Siegrist [43] could be aimed at clarifying the associations between work-related stress and fatigue. In addition, the cross-sectional study design could not clarify the developmental process and the causal relationship between prolonged fatigue and related factors. That is to say, high job demand and low job control could be the results as well as the causes of prolonged fatigue. Future research adopting a longitudinal study design in this field is recommended.

## Supporting Information

S1 Dataset The dataset of this study. (XLS)
